# Partogram of Grandmultiparous Parturients: A Multicenter Cohort Study

**DOI:** 10.3390/jcm12020592

**Published:** 2023-01-11

**Authors:** Joshua Guedalia, Michal Lipschuetz, Asnat Walfisch, Sarah M. Cohen, Eyal Sheiner, Abraham O. Samson, Joshua Rosenbloom, Doron Kabiri, Hila Hochler

**Affiliations:** 1 Department of Obstetrics and Gynecology, Hadassah Medical Center, Faculty of Medicine, Hebrew University of Jerusalem, Jerusalem 91121, Israel; 2 Department of Obstetrics and Gynecology, Rabin Medical Center, Petach-Tikva 49414, Israel; 3 Department of Obstetrics and Gynecology, Soroka University Medical Center, School of Public Health, Faculty of Health Sciences, Ben-Gurion University of the Negev, Beer Sheva 84101, Israel; 4 Faculty of Medicine, Bar Ilan University, Safed 13115, Israel

**Keywords:** grand multipara, multipara, cervical dilation, labor curve, partogram, progression rate, active phase, second stage

## Abstract

Sparse and conflicting data exist regarding the normal partogram of grand-multiparous (GMP, defined as parity of 6+) parturients. Customized partograms may potentially lower cesarean delivery rates for protraction disorders in this population. In this study, we aim to construct a normal labor curve of GMP women and compare it to the multiparous (MP, defined as parity of 2–5) partogram. We conducted a multicenter retrospective cohort analysis of deliveries between the years 2003 and 2019. Eligible parturients were the trials of labor of singletons ≥37 + 0 weeks in cephalic presentation with ≥2 documented cervical examinations during labor. Exclusion criteria were elective cesarean delivery without a trial of labor, preterm labor, major fetal anomalies, and fetal demise. GMP comprised the study group while the MP counterparts were the control group. A total of 78,292 deliveries met the inclusion criteria, comprising 10,532 GMP and 67,760 MP parturients. Our data revealed that during the first stage of labor, cervical dilation progressed at similar rates in MPs and GMPs, while head descent was a few minutes faster in GMPs compared to MPs, regardless of epidural anesthesia. The second stage of labor was faster in GMPs compared to MPs; the 95th percentile of the second stage duration of GMPs (48 min duration) was 43 min less than that of MPs (91 min duration). These findings remained similar among deliveries with and without epidural analgesia or labor induction. We conclude that GMPs’ and MPs’ cervical dilation progression in the active phase of labor was similar, and the second stage of labor was shorter in GMPs, regardless of epidural use. Thus, GMPs’ uterus function during labor corresponds, and possibly surpasses, that of MPs. These findings indicate that health providers can use the standard partogram of the active phase of labor when caring for GMP parturients.

## 1. Background

Determining whether labor is progressing normally is a key component of intrapartum care. Recognizing abnormal labor progression and intervening appropriately during protracted labor are crucial in reducing the risk of maternal and neonatal morbidity. In his milestone studies of the 1950s, Emanuel Friedman described criteria for the normal progress of labor and introduced a partogram of the mean, 5th, and 95th percentiles of cervical dilation over time [1,2]. A decade ago, these criteria were revised by Zhang and colleagues [3]. These labor curves, however, do not distinguish between MPs (parity of 2 to 5) and GMPs (parity of 6+), and no customized partogram is available for the latter population.

Failure to progress during labor is the leading indication for primary cesarean delivery, and in the United States alone, 35% of primary cesarian deliveries (CD) are performed due to protraction disorders [4].

Earlier studies found that different subgroups of parturients progress differently during labor, e.g., twin labors [5]. Customized partograms for different subgroups of parturients may potentially lower the rates of CDs due to protraction disorders.

Notably, the first labor progresses slower than all following labors [3], but whether high parity influences the cervical progression rate during labor is unknown. Histologic data are lacking regarding uterine morphological and functional changes after a few labors.

Data are also scarce about the labor progression rate among GMPs compared to MPs. We found only four studies [6,7,8,9] that were based on small cohorts and had conflicting results. Petrikovsky et al. studied 500 GMP deliveries without a control group and found that the active phase of labor begins at 5–6 cm of cervical dilatation and there is no deceleration phase [6]. Gurewitsch and coauthors included 1095 GMPs that exhibited a longer initial phase of labor and a similar curve as multiparas after reaching 6 cm [7]. Juntunen et al. reported no difference in the active phase duration when comparing nulliparous, para II, and para III but noted that GMP patients had shorter labors as a whole. However, each group consisted of only 42 parturients [8]. On the other hand, Lyrenäs et al. found a longer active phase once parity exceeded four, based on 272 GMPs deliveries [9]. Notably, all these studies collected data from labors occurring in the 1980s and 1990s.

In this study, we aimed to construct a normal partogram of GMP parturients in a large contemporary cohort and compare it to the MP labor curve in order to determine whether cervical dilation rates progress differently and whether medical interference during labor should be considered at a different point of time. Additionally, we aimed to separately analyze the partograms of subgroups with and without epidural analgesia and the induction of labor.

## 2. Methods

We conducted a multicenter retrospective cohort study in three tertiary medical centers in Israel: the obstetrics departments of (1) the Mt. Scopus and (2) Ein-Kerem campuses of Hadassah University Medical Center and of (3) Soroka University Medical Center. We reviewed the electronic medical records of deliveries occurring between the years 2003 and 2019 and created a single combined database.

The two campuses of Hadassah University Medical Center serve half of Jerusalem’s population of more than 1,160,000. Soroka University Medical Center is the sole hospital in the Negev region (southern Israel), serving a population of more than 1,000,000. Together, these medical centers serve ~20% of Israel’s population. The Israeli population is characterized by positive immigration and is therefore very diverse and multi-ethnic. Additionally, the three medical centers participating in this study are characterized by high delivery volumes as well as a large proportions of GMP parturients.

The study group included deliveries of GMP women (defined as parity of six and higher), while deliveries of parous women (defined as parity of two to five) comprised the comparison group. In order to give a complete overview of the partograms in our population, we also included labor data of nulliparous parturients in the tables and graphs. However, the statistical analysis was only performed for GMP versus MP parturients owing to the well-characterized slower progression of labor in nulliparas (NP) compared to any higher parity [3].

The inclusion criteria were a trial of labor of a singleton fetus, gestational age ≥37 weeks, cephalic presentation, and ≥2 documented cervical examinations during labor. The exclusion criteria were elective cesarean delivery (CD) without a trial of labor, the presence of a contraindication to vaginal delivery, non-vertex presentation, preterm labor, fetal demise, and deliveries in which cervical dilation was recorded less than twice. For the analysis of the length of only the second stage, labors ended by CD were also excluded because it inherently shortens the length of the second stage.

This study obtained ethical approval from the institutional review boards of Hadassah Hebrew University Medical Center (0130-22-HMO) and Soroka Medical Center (0153-22-SOR).

The perinatal database consisted of information recorded by an obstetrician immediately following delivery. Coding was performed according to the ICD-9 codes for all medical diagnoses after assessing medical prenatal care records as well as routine hospital documents. Data were collected in a blinded fashion by research staff members who were not involved in any stage of the perinatal care. The information was anonymized and de-identified prior to all analyses. Detailed information on maternal obstetric characteristics, medical history, reproductive and prenatal history, labor and delivery course and outcome, postpartum stay, and newborn information was obtained. Data on labor progression included repeated, time-stamped, cervical dilation and fetal-head station measurements. Notably, measuring cervical dilatation is a subjective method of examination, especially when not performed by the same person. In the participating medical centers, obstetricians and midwives examined the parturients during labor.

The induction of labor with oxytocin and augmentation were performed for GMPs using a low-dose protocol that started with 1 mU/min and elevated by 1 mU/min every 20–30 min, while for NPs and MPs the protocol started with 2 mU/min and elevated by 2 mU/min every 20–30 min.

The active phase of the first stage of labor was defined as the duration between cervical dilations of 6 and 10 cm, as the Consortium on Safe Labor data suggested that the active phase often begins at 6 cm and argued that active phase protraction, or labor arrest, should not be diagnosed before dilation of 6 cm [10].

Active phase duration was calculated from the last recorded dilation (time = 0) and backwards to the previous documented dilation(s) measurements, in hours (e.g., 1 h before complete dilation is −1 h). At the end of this calculation, the time was reverted to a positive value (i.e., −12 became 0 h, and 0 became 12 h).

The duration of the second stage of labor was calculated from the time of full dilation until delivery, in minutes. 

We calculated the durations of the active phase and second stage as the median (50% percentile) and 95th percentile of the group.

Traverse times were calculated as the time elapsed from one integer of cervical dilation in centimeters to the next. These centimeter-by-centimeter measurements of cervical dilation in labor were extrapolated from cervical examinations performed during labor. Missing values of cervical dilation, for those with a single or double consecutive gap in the examination recordings, were taken as the mean time of the available examinations.

In order to use fewer imputations and avoid making assumptions regarding the unknown cervical dilation between documented measurements, missing values of cervical dilation were replaced only for those with a single or double consecutive gap in the examination recordings. If two or more cervical examinations were available, the data were included in the analysis of the traverse times, regardless of the mode of delivery and whether full dilation was reached.

Customized partograms were constructed for different subgroups of parturients based on cervical dilation data, including parity, epidural analgesia use, and labor induction. 

*Statistical analysis*. The statistical analysis was performed using a Python script (3.7.3)^17^ and SPSS 27 for Windows (IBM Corp, Armonk, NY, USA). Categorical variables were analyzed with the χ^2^ test. Student’s *t*-test or the Mann–Whitney U test were applied to analyze differences in continuous variables, as appropriate. All graphs were generated using Matplotlib [11].

## 3. Results

During the study period, a total of 78,292 deliveries met the inclusion criteria. Of these, 10,532 deliveries of GMP women (sixth labor and higher) were identified and compared with 65,760 deliveries of MP women (second to fifth delivery). In order to provide a complete overview of the population, data regarding 39,934 nulliparous women are also shown. 

The clinical and demographic characteristics of the groups are presented in Table 1. As expected, GMP parturients were older, opted for epidural analgesia less frequently, and had a higher proportion of previous CD compared to MPs. In our cohort, GMPs experienced less postpartum hemorrhages (PPH) than MPs (1.1% vs. 1.7%, respectively, *p* < 0.001).

Table 2 shows the duration of the active phase of labor and each traverse time (the time elapsed from one integer of cervical dilation in centimeters to the next) in deliveries of GMPs and of MPs.

Remarkably, from 4 to 7 cm, the cervical dilation of GMPs progressed more slowly than MPs. The median of each traverse time was only a few minutes longer in GMPs. However, the 95th percentile durations were prolonged by 63 (4–5 cm), 30.5 (5–6 cm), and 18 (6–7 cm) minutes, growing shorter as the cervical dilation increased (Table 2).

Between 7 and 8 cm of dilation, there was no statistical difference between the groups. 

**Figure 1:**
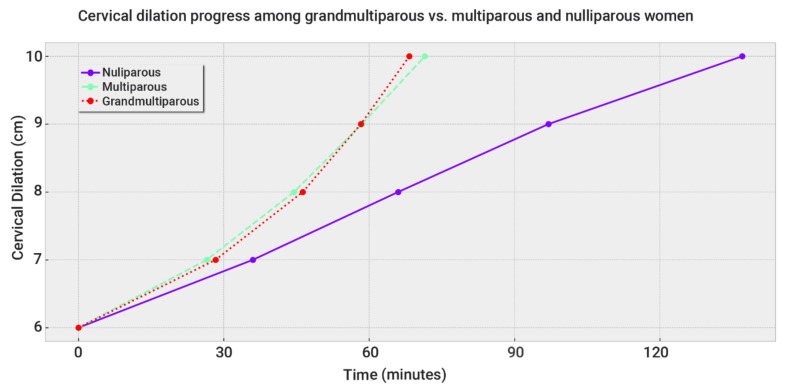
Partogram of GMPs, MPs, and NPs. As a whole, cervical dilation progression was very similar in grand- and multiparas, in contrast to the slow progression of nulliparas.

From 8 to 10 cm of cervical dilation, GMPs progressed faster than MPs. However, the duration difference of the 95th percentile was less than 10 min. Figure 1 plots these data as a partogram.

Taken together, the total median duration of the active phase of GMPs was 5 min shorter than MPs, while the 95th percentile was 6.5 min longer. This indicates that these small differences are of no clinical consequence.

**Figure 2:**
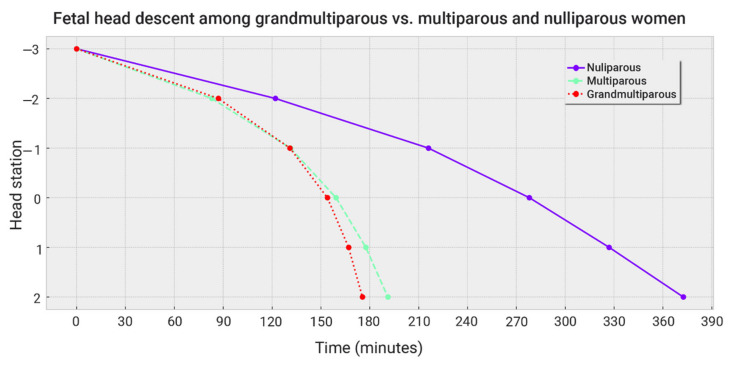
The slightly faster head descent of GMPs compared to MPs.

Figure 2 plots fetal head descent among GMP, MP, and NP women. Head descent was a few minutes faster in GMPs compared to MPs.

Table 3 summarizes the delivery modes of each group. The rates of unplanned CD were similar between GMPs and MPs and were low in all three groups (1.9% for GMPs and MPs, and 6.8% for NPs). GMPs were delivered less frequently via vacuum extraction (2% vs. 3.6% of MPs).

Notably, the main indications for CD did not differ among the groups (Table 4).

Table 5 presents data regarding the active phase duration for different subgroups of parturients with and without epidural analgesia or labor induction. In most of the subgroups, the active phase duration did not differ between GMPs and MPs. Only the subgroup of spontaneous deliveries without induction showed statistical significance. However, the difference was less than 10 min, and the trend of this difference was inverse at the median and the 95th percentile, indicating that it was clinically negligible.

Separately exploring labors with and without epidurals (Table 5) revealed no difference between the active phase durations of GMPs and MPs. The increased rate of epidural use in MPs compared to GMPs may have shifted the labor curve of MPs to a slower pace. Unfortunately, we could not adjust for the epidural rate.

Remarkably, the second stage of labor was shorter in GMPs compared to MPs (Table 6). The 95th percentile was 43 min longer in MPs (91 min) compared to GMPs (48 min). This was also true in women with epidural analgesia as well as with or without the induction of labor. 

## 4. Discussion

This multicenter study demonstrates that, compared to MPs, GMPs have a longer latent phase, a similar progression rate in the active phase, and a shorter second stage of labor. 

A worldwide effort is underway in recent years to decrease CD rates in order to prevent maternal and neonatal repercussions [10,12]. Failure to progress during labor is the leading indication for primary cesarean delivery in the USA. Therefore, there is a desire to promote vaginal birth and decrease the cesarean delivery rate while maintaining the safety of the woman and her fetus. Thus, in line with the worldwide effort to decrease CD rates, the use of customized partograms for different groups of parturients is essential.

Previous studies regarding labor progression among GMPs are sparse. They were based on small cohorts and found conflicting results. The largest study, by Gurewitsch et al. [7], examined 1590 GMPs, 1174 MPs, and 908 NPs during the years 1990–1995. The authors included only spontaneous labors, without inductions, of vertex fetuses at 36 + 0 weeks of gestational age or more. They found that GMPs progressed more slowly until 6 cm of cervical dilation and similar to MPs thereafter. Our larger cohort concurred with the slower progression until 6 cm but implied a faster rate from 8 cm onward.

Moreover, we divided our cohort to subgroups with and without epidural analgesia and the induction of labor. Stratifying parturients according to epidural use revealed no difference between the active phase durations of GMPs and MPs. Nevertheless, our data show that the 95th percentile duration of the active phase of induced labors of GMPs (321 min) was more than an hour longer than that of MPs (254 min) (Table 5). Excluding the induction of labors shifted the latent phase labor curve toward a faster progression and missed information regarding labors with induction. 

Furthermore, all previous studies [6,7,8,9] did not examine the second stage of labor, which we found to be 43 min shorter in GMPs’ labors. This was also true for women with epidural analgesia as well as with or without the induction of labor. Our data show that the 95th percentile of the second stage duration of GMPs was 48 min, almost half that of MPs (91 min).

Petrikovsky and colleagues [7] compared a partogram based on 500 GMPs to that of Friedman, without any control group, using labors that occurred between the years 1978 and 1981. The exclusion criteria were wide: abnormal antepartum features (no details provided); abnormal intrapartum features, including the induction or augmentation of labor; and those with operative termination. They also excluded labors that resulted in compromised infants: 1 min Apgar < 7, 5 min Apgar < 8, and infants weighing < 2700 and >4200 g. They found that GMPs have a biphasic, exponential labor curve. The active phase of labor begins at 5–6 cm of cervical dilatation. There is no deceleration phase, and head descent begins at 7–8 cm of cervical dilatation. The authors did not test their own model against MP control subjects and therefore could not compare them. Furthermore, the exclusion criteria in the two studies differed markedly, as Friedman included patients with operative deliveries, augmented labor, and poor outcomes. 

Finally, another study by Lyrenäs and colleagues, which was based on a small cohort of 242 deliveries [9], demonstrated contradicting results, with a longer active phase once parity exceeded four.

Our study revealed that GMPs’ uterus function during labor corresponds to that of MPs. This is supported by recent studies from high-resource countries, which found that GMPs have a similar risk for uterine rupture compared to MPs [13,14,15,16,17]. A recent study that was based on a large cohort of 386,019 GMPs revealed that, in adjusted analyses, women with grand multiparity were at lower risk of severe maternal morbidity (aRR 0.93, 95%CI: 0.89, 0.96) [18]. This is reflected in clinical practice by the fact that oxytocin is an accepted method for labor augmentation among grand-multiparous women [19].

## 5. Conclusions

The latent phase of GMPs is a few minutes longer than that of MPs, while the active phase is similar in length. The second stage of labor of GMPs is 43 min shorter than that of MPs, both with and without epidural analgesia. These findings indicate that the standard partogram of the active phase of labor is suitable for GMP women. Finally, GMPs’ uterus function during labor corresponds, and possibly surpasses, that of MPs.

## 6. Clinical Implications

We did not find any data regarding a histologic difference between GMP and MP uteri. Clinically, our data show that GMP uterus function is similar to that of MPs during the active phase and faster in the second stage. This indicates that health providers can rely on the standard partogram of the active phase of labor for GMP parturients.

## 7. Strengths and Limitations

The strengths of our study are the large cohort of GMPs and delivery in medical centers characterized by modern obstetrics. Our diverse and multi-ethnic populations enable the application of the results to other populations. We studied all phases of labor, including the second stage of labor, which had not been studied.

The main limitation of our study is its retrospective design. However, all previous studies regarding partograms were retrospective. Furthermore, prospective studies are also needed to validate our partograms in other populations. Additionally, measuring cervical dilatation is a subjective method of examination, especially when not performed by the same person. In the participating medical centers, obstetricians and midwives examined the parturients during labor.

## Figures and Tables

**Table 1:** Obstetric parameters of parturients with twins and singleton deliveries.

	Grandmultipara (GMP)	Multipara (MP)	Nullipara (NP)	*p*-Value *
Participants (*n*)	10,532	65,760	39,934	
Age in years (median, IQR)	35 (32–38)	29 (26–33)	25 (22–29)	<0.001
Parity (median, IQR)	6 (5–7)	2 (1–3)	-	<0.001
Previous CD (*n*, %)	1924 (15.4%)	7940 (10.8%)	-	<0.001
Gestation week (median, IQR)	40 (39–41)	40 (39–41)	40 (39–41)	<0.001
Induction (*n*, %)	1430 (11.5%)	8489 (11.5%)	7280 (18.2%)	0.914
Epidural analgesia (*n*, %)	3958 (31.8%)	35,250 (47.8%)	27,071 (67.8%)	<0.001
3rd and 4th degree perineal tears (*n*, %)	2 (0.0%)	74 (0.1%)	262 (0.7%)	0.002
Postpartum hemorrhage (*n*, %)	137 (1.1%)	1238 (1.7%)	762 (1.9%)	<0.001
Neonatal birth weight (grams) (median, IQR)	3440 (3145–3736)	3336 (3065–3618)	3200 (2940–3470)	<0.001
Apgar at 5 min ≤ 7 (*n*, %)	46 (0.4%)	163 (0.2%)	324 (0.8%)	0.002

* Chi square or Mann–Whitney U test comparing only MP and GMP groups.

**Table 2:** Traverse times of cervical dilation progression.

Cervical Dilation in Centimeters	Grandmultipara (GMP)	Multipara	Nullipara	*p*-Value **
		(MP)	(NP)	
4–5	74.7 (364.0) *	68.0 (301.0) *	86.0 (368.0) *	<0.001
5–6	42.5 (194.0) *	38.7 (163.5) *	51.0 (202.0) *	<0.001
6–7	28.0 (129.0) *	26.5 (111.0) *	36.0 (137.0) *	<0.001
7–8	17.7 (89.0) *	18.0 (80.7) *	30.0 (108.0) *	0.865
8–9	12.0 (72.0) *	14.0 (68.5) *	31.0 (112.0) *	<0.001
9–10	10.0 (61.3) *	13.0 (71.0) *	40.0 (148.0) *	<0.001
**6–10**	58.0 (261.5) *	63.0 (255.0) *	143.0 (401.0) *	<0.001

* Median time in minutes (95th percentile). ** Mann–Whitney U test comparing only GMP vs. MP.

**Table 3:** Delivery mode.

	Grandmultipara (GMP)	Multipara (MP)	Nullipara (NP)	*p*-Value	Odds Ratio (95% CI) *
Vaginal delivery (*n*, %)	11,971 (96.1%)	696,764 (94.5%)	30,864 (77.3%)	Reference group	
Instrumental delivery (*n*, %)	248 (2.0%)	2660 (3.6%)	6366 (15.9%)	<0.001	0.54 (0.48–0.62)
Unplanned cesarean (*n*, %)	237 (1.9%)	1367 (1.9%)	2704 (6.8%)	0.975	1.01 (0.87–1.15)

* GMP vs. MP.

**Table 4:** Indications for cesarian delivery (CD).

	Grandmultipara	Multipara	Nullipara	*p*-Value *
	(GMP)	(MP)	(NP)	
Dysfunctional labor (*n*, %)	84 (36.5%)	567 (42.4%)	1466 (54.7%)	0.288
Fetal stress (*n*, %)	104 (45.2%)	647 (48.4%)	978 (36.5%)	0.673
Maternal stress (*n*, %)	6 (2.6%)	16 (1.2%)	29 (1.1%)	0.119
Other (*n*, %)	36 (15.7%)	108 (8.1%)	207 (7.7%)	<0.001

* GMP vs. MP.

**Table 5:** Duration of active phase of first stage of labor.

	Grand Multipara (GMP)	Multipara (MP)	Nullipara (NP)	*p*-Value **
All	58.0 (261.5) *	63.0 (255.0) *	143.0 (401.0) *	<0.001
Epidural	87.0 (319.0) *	84.0 (288.0) *	157.0 (411.0) *	0.124
No Epidural	46.0 (223.0) *	45.0 (206.0) *	107.0 (372.0) *	0.522
Induction	52.5 (321.0) *	50.0 (254.0) *	144.0 (405.0) *	0.861
No Induction	59.0 (259.0) *	65.0 (255.0) *	143.0 (400.0) *	<0.001

* Median time in minutes (95th percentile), ** Mann–Whitney U test comparing GMP vs. MP.

**Table 6:** Duration of second stage of labor.

	Grand Multipara (GMP)	Multipara (MP)	Nullipara (NP)	*p* Value **
All	8.0 (48.0) *	11.0 (91.0) *	79.0 (203.0) *	<0.001
Epidural	10.0 (69.0) *	17.0 (115.0) *	97.0 (207.0) *	<0.001
No Epidural	6.0 (34.0) *	8.0 (46.0) *	43.0 (170.0) *	<0.001
Induction	8.0 (54.0) *	13.0 (102.0) *	93.0 (211.0) *	<0.001
No Induction	7.0 (47.0) *	11.0 (89.0) *	75.0 (200.0) *	<0.001

* Median time in minutes (95th percentile), ** Mann–Whitney U test comparing GMP vs. MP.

## Data Availability

Not applicable.
